# Altered Expressions of miR-1238-3p, miR-494, miR-6069, and miR-139-3p in the Formation of Chronic Brucellosis

**DOI:** 10.1155/2016/4591468

**Published:** 2016-09-19

**Authors:** Ferah Budak, Salih Haldun Bal, Gulcin Tezcan, Halis Akalın, Guher Goral, Haluk Barbaros Oral

**Affiliations:** ^1^Department of Immunology, Faculty of Medicine, Uludag University, Bursa, Turkey; ^2^Department of Medical Biology, Faculty of Medicine, Uludag University, Bursa, Turkey; ^3^Department of Infectious Diseases and Clinical Microbiology, Faculty of Medicine, Uludag University, Bursa, Turkey; ^4^Department of Medical Microbiology, Faculty of Medicine, Uludag University, Bursa, Turkey

## Abstract

Brucellosis is a zoonotic disease that is still endemic in developing countries. Despite early diagnosis and treatment of patients, chronic infections are seen in 10–30% of patients. In this study, we aimed to investigate the immunological factors that play roles in the transition of brucellosis from acute infection into chronic infection. Here, more than 2000 miRNAs were screened in peripheral blood mononuclear cells (PBMCs) of patients with acute or chronic brucellosis and healthy controls by using miRNA array, and the results of the miRNA array were validated through qRT-PCR. Findings were evaluated using GeneSpring GX (Agilent) 13.0 software and KEGG pathway analysis. Four miRNAs were expressed in the chronic group but were not expressed in acute and control groups. Among these miRNAs, the expression level of miR-1238-3p was increased while miR-494, miR-6069, and miR-139-3p were decreased (*p* < 0.05, fold change > 2). These miRNAs have the potential to be markers for chronic cases. The differentially expressed miRNAs and their predicted target genes involved in endocytosis, regulation of actin cytoskeleton, MAPK signaling pathway, and cytokine-cytokine receptor interaction and its chemokine signaling pathway indicate their potential roles in chronic brucellosis and its progression. It is the first study of miRNA expression analysis of human PBMC to clarify the mechanism of inveteracy in brucellosis.

## 1. Introduction

Brucellosis is a zoonotic disease spreading from animals to humans and is observed endemically in developing countries. It especially affects domestic animals like sheep, goat, buffalo, and steer and spreads from these animals to humans through direct or indirect routes of transmission thus creating a significant burden on public health resources.

Even though the disease is seen all over the world, it is especially seen in Mediterranean countries including Turkey along with Arabia, India, Mexico, and Central and South America [1]. There are* Brucella* bacteria in urine, milk, placenta, and other secretions of sick animals. They mostly spread by cuts and wounds on the skin as well as direct contact with the infected animal or its secretions, inhalation of infected aerosols, or inoculation to the conjunctiva, as well as via gastrointestinal system with the consumption of unpasteurized milk or dairy products [2, 3].* Brucella* species are intracellular facultative bacteria that are 0.6–1.5 *μ*m long; they are Gram-negative, do not form spores, and live in macrophages [4]. The majority of human cases are caused by* Brucella melitensis*, although* Brucella abortus*,* Brucella suis*,* Brucella canis*, and more recently* Brucella pinnipedialis* and* Brucella ceti* have also been associated with human disease [5].* B. melitensis* accounts for the majority of the brucellosis cases in humans in Turkey [6]. Symptoms such as fever, fatigue, loss of appetite, headache, back ache, weight loss, myalgia, and arthralgia are observed in acute brucellosis [7]. The acute phase of brucellosis follows the eradication of the bacteria by the immune system or the infection transitions into the chronic form defined by mild fever, sweating, weight loss, and localized infections which resemble chronic fatigue syndrome. Chronic infection is seen in about 10–30% of the patients despite early diagnosis and treatment [2, 8]. The diagnosis of chronicity is mainly based on clinical symptoms and findings together with the presence of high immunoglobulin G (IgG) titers determined by serological tests [9]. However, serological assays maintain low specificity when it comes to diagnosis of brucellosis because IgG titers may remain positive for years following the successful resolution of symptoms [2, 10]. Thus, these facts suggest that good markers for clinical prediction, diagnosis, and follow-up of brucellosis are needed to provide effective and accurate treatment regiments. Additionally, mechanisms leading to chronicity are not completely established. Therefore, understanding of mechanisms involved in evasion from host immune responses will provide valuable information about the pathogenesis of brucellosis and shed light on the development of new treatment strategies for the treatment of brucellosis and the prevention of transition to chronic form.

Some studies demonstrated that* Brucella* bacteria can evade from immune response to establish persistent and chronic infection [11]. Most prominent immune evasion mechanisms of* Brucella* include inhibition of complement system and TLR signaling pathways, disruption of effective antigen presentations to CD4+ T cells, impairment of dendritic cell activation, selective subversion of autophagy pathways, and inhibition of macrophage apoptosis and autophagolysosomal fusion [12–19]. Also, recent studies done on* in vitro* models shed light on the potential roles of miRNAs for immune evasion mechanisms used by* Brucella* [20, 21].

MicroRNA (miRNA) is a small RNA set with approximate dimensions of about 22 nt. miRNA forms about 1–4% of the RNA in human genome. It targets messenger RNA to carry out important regulatory functions. A single miRNA can regulate about 200 messenger RNA functions. It becomes active by breaking up the target messenger RNAs or by suppressing its translation [22, 23]. miRNAs take part in many functional developments and diseases such as hematopoiesis regulation, cellular proliferation, cell differentiation, organogenesis, apoptosis, cancer development, infection development, and heart diseases [24–30].

The objectives of our study were to investigate the miRNA expression changes of the peripheral blood mononuclear cells (PBMCs) of brucellosis patients that may play important roles in the clearance and chronicity of the* Brucella* infection. To find out which of the miRNAs affects the transition into the chronicity, the expression changes of the miRNAs involved in immune responses of PBMCs were examined in acute and chronic brucellosis and healthy control groups.

## 2. Material and Method

### 2.1. Inclusion of Participants

Participants were 16 patients with acute (3 female, 7 male, total: 10) or chronic (3 female, 3 male, total: 6) brucellosis diagnosis accompanied with bone and joint involvement who have applied to the Uludag University, Faculty of Medicine, Clinical Bacteriology and Infection Diseases Department. Seven healthy controls (3 female, 4 male) were included in the study. The cases were classified according to the onset time of the symptoms as acute (0–2 months) and chronic (>12 months) [1, 31]. Brucellosis diagnosis was made with the existence of one or more constitutional symptoms such as fever, perspiration, and fatigue and the standard tube agglutination test titer being ≥1/160 [7]. Patients were treated with doxycycline 100 mg PO twice daily and rifampicin 600–900 mg PO daily for 45 days [32]. This study protocol was approved by the Ethics Committee of the University of Uludag, Faculty of Medicine (Permit number: 2010-6/2).

### 2.2. RNA Isolation

PBMCs were separated from the 20 mL blood samples obtained from the patients in acute, chronic, and healthy control groups via intensity gradient centrifuge method with Ficol (Biochrom, Germany). Cells were stored with TriPure Isolation Reagent (Roche, Germany) at −80°C until use. miRNeasy Kit (Qiagen, Germany) was used in accordance with the manufacturer protocol for the total RNA isolation from the isolated PBMCs. The measurements of concentrations and purities were carried out using the Nanodrop (Thermo Scientific, USA) equipment. RNA qualities were determined in Agilent Bioanalyzer (Agilent, UK) device using Agilent RNA 6000 Nano Kit.

### 2.3. miRNA Microarray Analysis

MicroRNAs from RNA samples were marked with Cy3 fluorescent dye by using miRNA Labeling Kit and Spike Kit (Agilent, UK). MicroRNA Hybridization Kit (Agilent, UK) was used to hybridize into Human miRNA Microarray, Release 19.0, 8 × 60 K (v19) microarray slides (Agilent, UK) and scanned using Nimblegen MS200 array scanner. The TIFF image files obtained were processed using Agilent Feature Extraction software to extract raw data and obtain QC reports. The data of the samples that pass QC parameters were subject to quantile normalization and analyzed using GeneSpring GX (Agilent, UK) 13.0 software after which microRNAs with *p* values of >0.05 and fold change values of >2 and <−2 were determined to be statistically significant.

### 2.4. miRNA Validation with qRT-PCR Analysis

For validation of miRNA expression, Universal cDNA Synthesis kit (Exiqon, Denmark) was used for cDNA synthesis. Housekeeping gene (*SNORD48*) and cDNA control were controlled with a spike in primers. Of the results of cDNA's Snord48 and Spike, those that are between Ct values 15 and 29 regarding Ct values were worked in LightCycler 480 II (Roche, Germany) using microRNA LNA*™* primer sets (Exiqon, Denmark) and ExiLENT SYBR® Green Master Mix kit for the specified miRNAs. Δ/ΔCt method was used to carry out relative quantification for the acquired results. With this method, miRNA Ct values were normalized via Snord48 and U6 housekeeping genes (this value is denoted as ΔCt) after which the groups were compared with themselves and the acquired result yielded Δ/ΔCt and if this value is greater than 2, regulation result was determined as a target positive expression increased and if it is less than −2, target down was determined as the result of being controlled by the microarray data.

### 2.5. KEGG Pathway Analysis of Target Genes

Pathway enrichment analysis was performed to understand the biological potency and functional classification of predicted miRNA targets. WebGestalt (WEB-based GEneSeTAnaLysis Toolkit) [33] (http://bioinfo.vanderbilt.edu/webgestalt/) web-based enrichment analysis tool was used for pathway analysis and KEGG (Kyoto Encyclopedia of Genes and Genomes) [34] (http://www.genome.jp/kegg/pathway.html) pathway enrichment was used to elucidate the pathways of the predicted miRNA target genes.

## 3. Results

While 10 of the 16 patients (3 female, 7 male) were diagnosed with acute brucellosis, 6 of the patients (3 female, 3 male) were diagnosed with chronic brucellosis. The median age at diagnosis of acute brucellosis was 51.5 ± 13.5 and at diagnosis of chronic brucellosis was 44.2 ± 12.4. Also, the median age of 7 healthy volunteers (3 female, 4 male) was 37.3 ± 5.7.

### 3.1. miRNAs Play Role in Acute and Chronic Brucellosis

**Figure 1:**
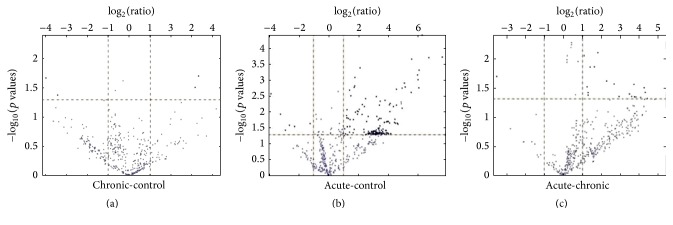
Differential expressions of miRNAs evaluated in (a) chronic versus control, (b) acute versus control, and (c) chronic versus acute cases depend on mRNA microarray analysis (*p* < 0.05, cut-off = 2). The volcano plot demonstrates the differential expression of the illustrated genes; dots in light represent genes that did not achieve significant changes in expression, dots in the dark on the left indicate the genes with significantly downregulated expression, and dots in the dark on the right indicate the genes with significantly upregulated expression.

miRNA microarray data of PBMC cells of acute and chronic brucellosis patients and control group were analyzed with GeneSpring GX 13.0 software (Agilent, UK). Because miRNAs with less than 2-fold alteration in expression were assumed as not significant, these miRNAs were ignored (Figure 1).

Five miRNAs were altered in all brucellosis cases when compared to control group. However, the expressions of these miRNAs did not show a significant difference between chronic and acute group (Table 1).

Moreover, not only were 15 miRNA expressions different from control group but also they displayed a difference between chronic and acute brucellosis cases (Table 2).

In comparison to acute cases, expression levels of 4 miRNAs were significantly altered in chronic cases. The expressions of these miRNAs were similar in acute cases and healthy controls (Table 3).

In addition, expression levels of 36 miRNAs were significantly altered in acute cases. The expression of these genes was similar for chronic cases and control group (Table 4).

### 3.2. Predicted Target Pathways of miRNA

**Figure 2:**
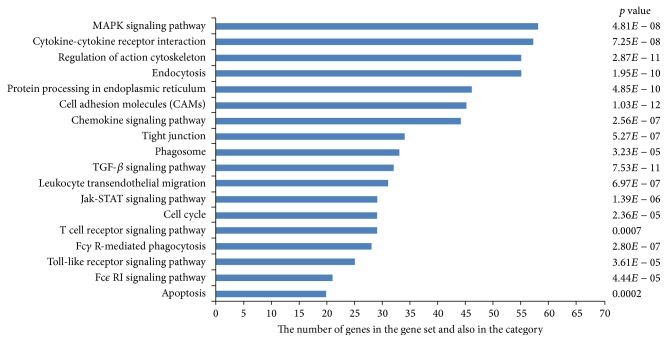
Pathway analysis of miR-1238-3p according to KEGG function annotations.

To understand the role of immunological and genetic factors involved in the transition of brucellosis into chronic infection, target pathway prediction of miR-1238-3p, miR-494, miR-6069, and miR-139-3p was performed according to KEGG function annotations, which increased miRNA (miR-1238-3p) of target genes involved in immunologically effective pathways as shown in Figure 2.

**Figure 3:**
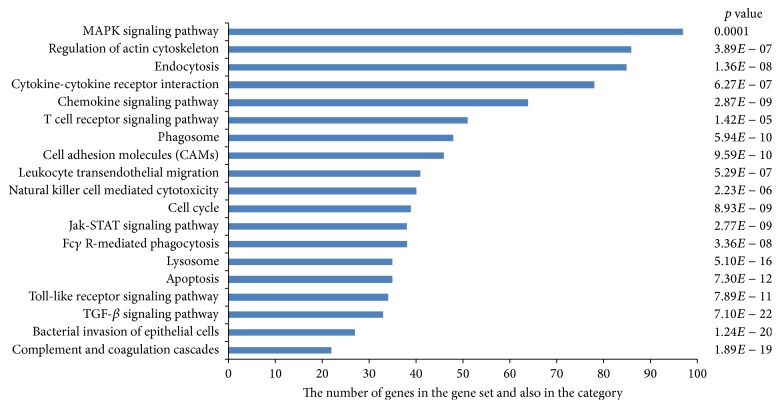
Pathway analysis of 3 downregulated miRNAs, miR-494, miR-139-3p, and miR-6069 according to KEGG function annotations.

**Figure 4:**
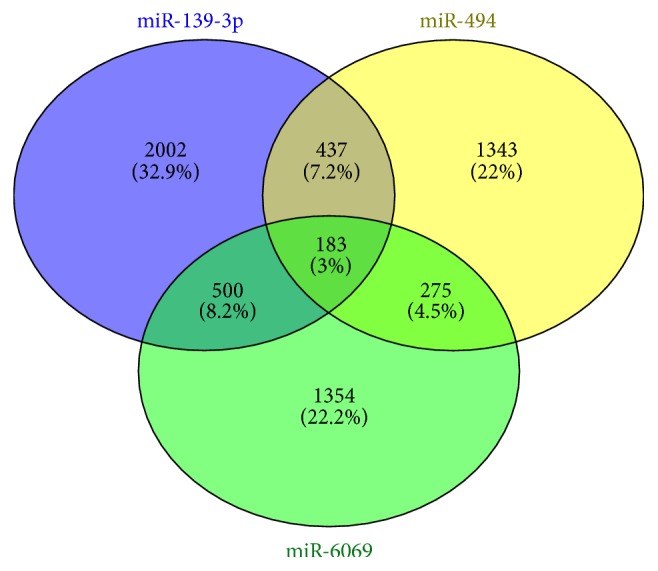
Target gene prediction for the 3 downregulated miRNAs, miR-494, miR-139-3p, and miR-6069.

miRNAs (miR-494, miR-6069, and miR-139-3p) that were downregulated in the chronic group were considered common. Results showed that about 7686 predicted genes were annotated, with the genes primarily active in endocytosis, regulation of actin cytoskeleton, MAPK signaling pathway, cytokine-cytokine receptor interaction, and chemokine signaling pathways associated with immunity (Figure 3). The predicted number of target genes is present in Figure 4.

**Figure 5:**
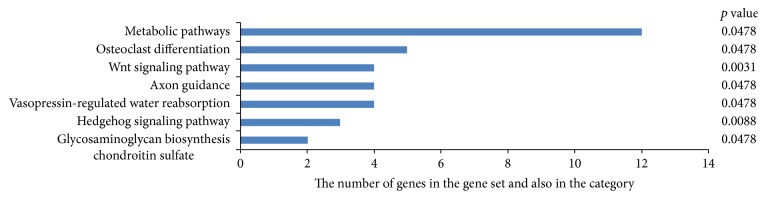
Common pathway analysis of miR-494, miR-139-3p, and miR-6069 according to KEGG function annotations.

183 genes (3%) were regulated mutually by miR-494, miR-139-3p, and miR-6069. According to KEGG function annotations, these mutual genes play a role in multiple signaling pathways, such as the metabolic pathways, osteoclast differentiation, Wnt signaling pathway, axon guidance, vasopressin-regulated water reabsorption, Hedgehog signaling pathway, and glycosaminoglycan biosynthesis chondroitin sulfate (Figure 5).

## 4. Discussion and Conclusion

Our understanding of the mechanisms of the transition from acute infection to chronic brucellosis remains incomplete. Multiple bacterial and host factors may be involved in this complex process [11].

Herein, we focused on miRNAs that play key roles in the fundamental cellular process. The expression profile of these miRNAs showed high variability between individuals and was independent of their age, gender, or clinical phenotype. In our study, we investigated the expression patterns of several miRNAs in PBMCs of chronic and acute cases and the control group to establish posttranscriptional regulation of gene expression and immunological basis involved in course of chronicity. Although there have been no studies investigating miRNA profiles in brucellosis, there are few studies investigating the differences in miRNA expression levels between acute and chronic viral infections in humans. Most of these studies are focused on circulating miRNAs in acute and chronic forms of Hepatitis or acute and chronic phases of Hepatitis C [35–37].

In this study, all patients received the same antibiotic regimen including doxycycline and rifampicin. In two studies, it was demonstrated that rifampicin may alter miRNA expression in human hepatocyte cultures [38, 39]. Apart from those studies, there are no studies available that display the role of antibiotics with regard to changes in miRNA expression in other cells including PBMCs* in vitro* or* in vivo*. Therefore, this issue was not considered in this study, since the same antibiotic regimen was used for all patients.

The findings discussed here reveal the first detailed snapshot of miRNA expression levels in PBMCs of brucellosis patients. The only study focused on miRNA expression associated with* Brucella* infections has been published by Zheng et al. and examined the miRNA expression profiles in RAW264.7 cells in response to* Brucella melitensis* infection. They reported 344 unique miRNAs which were coexpressed in the two libraries (mock- and* Brucella*-infected RAW264.7 cells) in which 57 miRNAs were differentially expressed. Eight differentially expressed miRNAs with high abundance were subjected to further analysis. The GO enrichment analysis suggests that the putative target genes of these differentially expressed miRNAs are involved in apoptosis, autophagy, and immune responses. In particular, a total of 25 target genes participate in regulating apoptosis and autophagy, indicating that these miRNAs may play important regulatory roles in the* Brucella*-host interactions [20]. In this current study, the regulatory role of 2000 miRNAs was evaluated in human PBMCs. We determined 5 miRNAs involved in* Brucella* infection, 15 miRNAs which were differently regulated between chronic and acute brucellosis, and 4 miRNAs uniquely expressed in chronic brucellosis.

### 4.1. Similarly Expressed miRNAs in Chronic and Acute Brucellosis

Five miRNAs (miRNA-575, miRNA-1914-3p, miRNA-339-5p, miRNA-22-5p, and miRNA-335-5p) displayed similar expression trends in both the chronic and acute infection groups compared with the control group, which indicated that both chronic and acute* Brucella* infections might share, at least partly, similar regulatory mechanisms.

### 4.2. Differently Expressed miRNAs in Chronic Brucellosis in Comparison to Acute Brucellosis

To identify miRNAs in PBMC that might be correlated with chronicity, we focused on the differentially expressed miRNAs between the chronic group and the acute infection group. According to our findings, there were 15 dysregulated miRNAs among the comparison groups. Whereas fourteen miRNAs were decreased, the expression of a miRNA, miR-125b-5p, increased in the chronic group compared with the acute infection group.

One of the downregulated miRNAs in chronic patients, miR-630, is involved in the apoptotic processes and pathways that dictate autophagy [40–42]. Cao et al. demonstrated that miR-630 inhibits cell proliferation by targeting cell-cycle kinase 7 (CDC7) but maintains the apoptotic balance by targeting multiple activators of apoptosis under genotoxic stress [43]. Numerous studies show that* Brucella* causes inhibition of macrophage apoptosis and activation to ensure its own intracellular growth within macrophages [19, 44].* Brucella* also leads to inhibition of synoviocyte apoptosis through the upregulation of antiapoptotic factors (cIAP-2, clusterin, livin, and P21/CIP/CDNK1A) [45]. Several studies indicated that* Brucella* has evolved strategies to protect itself against autophagy or to control the components of autophagy to its benefit [46]. On the other hand, according to Velásquez et al.* B. abortus* induces apoptosis of human T lymphocytes, even though the invasion of T lymphocytes was low and nonreplicative and* B. abortus* is directly inhibiting T cell-mediated responses and evade adaptive immune responses [47]. Although the previous findings imply the different effect of brucellosis in cell apoptosis in individual cells, the role of miRNA in this mechanism has not been taken into consideration in the previous studies. According to presented findings, dysregulation of miR-630 might be effective in brucellosis behavior on cell apoptosis and autophagy.

Seven of the downregulated miRNAs in chronic patients were found to be associated with carcinogenesis in various organs. While miR-1290 and miR-572 were found to be upregulated, miR-125a-3p, miR-134, miR-584-5p, miR-663a, and miR-513a-5p were determined to be downregulated in various types of cancer [48–64]. Among these miRNAs, miR-134 and miR-125a-3p have been linked to infectious diseases. Schnitger et al. demonstrated that miR-125a-3p take part in the early innate immune response of macrophages to* Listeria* infection [65]. According to Kumar and Nerurkar miR-125a-3p target multiple genes involving production of cytokines, chemokines, and expression of apoptotic genes, which belong to different signaling pathways that play a critical role in West Nile virus neuropathogenesis [66]. Additionally, the expression level of miR-125a-3p was determined in higher levels in HIV (+) samples than in healthy controls [67]. miR-134 was defined as elevated in patients infected with Hepatitis C virus compared to healthy controls [68]. Moreover, according to Gao et al., the dynamic expression of miR-134 has been increasing with time in infectious mononucleosis caused by primary Epstein-Barr virus [69]. Interestingly, the expression of this miRNA is uniquely presented in the current study where it was downregulated in patients with brucellosis infections.

On the other hand, although miR-211-3p, miR-1277-5p, miR-150-3p, miR-513b, miR-664b-5p, and hsa-miR-1285-3p were determined as downregulated in chronic brucellosis in the present study, the function of these miRNAs in infectious diseases remains unknown.

The increased miRNA in chronic patients, miR-125b-5p, is known to regulate various immune system functions. In a study of Ouyang et al., miR-125b-5p associate with inhibited cell proliferation and promote adipogenic differentiation [70]. In another study, Lu et al. showed that miR-125b-5p attenuated lipopolysaccharide-induced monocyte chemoattractant protein-1 production [71]. Chaudhuri et al. have demonstrated that miR-125b-5p modulate inflammatory chemokine CCL4 expression in immune cells and potentiate macrophage activation [72, 73]. A study carried out by Huang et al. demonstrated that miR-125b can negatively regulate TNF-*α* mRNA expression and protein synthesis in monocytes [74]. It was determined in our study that miR-125b-5p expression is elevated in PBMCs of the chronic group when compared with the acute infection group. Supporting previous data, our results reveal that the increased activity of the signal pathways may be involved in chronic brucellosis.

### 4.3. Specifically Altered miRNAs in Chronic Brucellosis

Describing the prognostic factors associated with chronicity of brucellosis infection is substantial for contributing to the follow-up treatment and improving alternative therapy protocols for these patients. In the current study, 4 miRNAs were determined to display a statistically significant change in chronic cases in comparison with both the acute cases and the control group. miR-1238-3p was upregulated, while miR-494, miR-6069, and miR-139-3p were downregulated in the chronic group compared with the active group.

Downregulated microRNAs, miRNA-494, and miR-139-3p have been proven to be involved in the carcinogenesis and development of various types of cancer in previous studies [75–80]. In the present study, miRNA-494 was linked to MAPK signaling pathway, regulation of actin cytoskeleton, chemokine signaling pathway, natural killer cell-mediated cytotoxicity, apoptosis, phagosome, T cell receptor signaling, Fc*γ*-R-mediated phagocytosis, cell-cycle, TGF-*β* signaling pathway, and complement and coagulation cascades in chronic brucellosis patients. miR-139-3p was also linked to endocytosis, regulation of actin cytoskeleton, cytokine-cytokine receptor interaction, MAPK signaling pathway, chemokine signaling pathway, cell adhesion molecules, phagosome, T cell receptor signaling pathway, leukocyte transendothelial migration, and bacterial invasion of epithelial cells pathways. The third downregulated miRNA was miR-6069. Although the function of this miRNA remains unknown in biological processes, in the current study we uniquely determined that reduced expression of miR-6069 may have functions in regulation of actin cytoskeleton, MAPK signaling pathway, endocytosis, cytokine-cytokine receptor interaction, chemokine signaling pathway, JAK-STAT signaling pathway, T cell receptor signaling pathway, leukocyte transendothelial migration Toll-like receptor signaling pathway, Fc*γ*R-mediated phagocytosis, and bacterial invasion of epithelial cells signaling pathways in chronic brucellosis.

Mutual KEGG pathway analysis of miR-494, miR-139-3p, and miR-6069 revealed that pathways related to these miRNAs in brucellosis have biological significance associated with the conversion of chronicity, including endocytosis, regulation of actin cytoskeleton, MAPK signaling pathway, cytokine-cytokine receptor interaction, and chemokine signaling pathways. Endocytosis and regulation of actin cytoskeleton pathway are very important for intracellular bacterial clearance. Inhibition of intracellular bacterial replication is related to its control of endocytosis and membrane fusion events between endosomes and* Brucella*-containing phagosomes. The MAPK cascade is one of the most ancient and evolutionarily conserved signaling pathways and is involved in all aspects of immune responses in mammalian hosts. This signaling cascade is activated by different PAMPs, which play an important role in the phagocytosis of bacteria and remodeling of the actin cytoskeleton [81, 82].

We also found 183 mutual genes (3%) that were regulated by the three downregulated miRNAs. Pathway analysis suggested that these mutual genes were involved in multiple signaling pathways. An important part of signaling pathways that were impacted due to differential expression of the discussed miRNAs during brucellosis were particularly important with regard to bone and joint development. Osteoarticular brucellosis is the most common presentation of the active disease in humans, affecting up to 85% of patients. The three most common forms of osteoarticular involvement are sacroiliitis, spondylitis, and peripheral arthritis. Loss of bone is a serious complication of localized bacterial infection of bones or the adjacent tissue.* B. abortus* may directly and indirectly harm osteoblast function, contributing to the bone and joint destruction observed in patients with osteoarticular complications of brucellosis [83]. In our study, all of the cases were composed of brucellosis patients with bone and joint involvement. Effective signaling pathways in the development of bone and joint were osteoclast differentiation, Wnt signaling pathway, Hedgehog (Hh) signaling pathway, vasopressin-regulated water reabsorption, and glycosaminoglycan biosynthesis chondroitin sulfate. Wnt signaling has been shown as an important regulatory pathway in the osteogenic differentiation of mesenchymal stem cells. Induction of the Wnt signaling pathway promotes bone formation while inactivation of the pathway leads to osteopenic states [84]. Hh also plays an important role in bone homeostasis, and reducing Hh signaling protects against age-related bone loss. Disruption of Hh signaling regulation leads to multiple bone diseases, such as progressive osseous heteroplasia. Interactions between Hh and Wnt signaling regulate cartilage development, endochondral bone formation, and synovial joint formation [85]. In the kidney, the antidiuretic hormone vasopressin (AVP) is a critical regulator of water homeostasis by controlling the water movement from lumen to the interstitium for water reabsorption and adjusting urinary water excretion. Hyponatremia was known to be associated with osteoporosis and a high fracture risk. Hyponatremic patients have elevated circulating AVP levels [86]. Glycosaminoglycans are polysaccharides made up of repeating disaccharide units and are an important component of many connective tissues including tissues in the equine joint. The most important GAGs in the joint are chondroitin sulfate, hyaluronic acid, and keratin sulfate [87]. Chondroitin sulfate is an important structural component of cartilage and provides much of its resistance to compression [88]. Loss of chondroitin sulfate from cartilage is one of the main causes of osteoarthritis.

The only upregulated miRNA in chronic brucellosis was miR-1238-3p. Despite the absence of a clear explanation for the function of this miRNA, the present study displayed the effects of altered expression of this miRNA on MAPK signaling pathway, cytokine-cytokine receptor interaction, regulation of actin cytoskeleton, endocytosis, protein processing in endoplasmic reticulum, cell adhesion molecules, and chemokine signaling pathway in chronic brucellosis through KEGG pathway analysis.

In conclusion, identifying the prognostic factors for chronicity is required for follow-up treatment and improving alternative therapy protocols in brucellosis infection. In the present study, we uniquely determined that reduced expression of miR-139-3p, miR-6069, and miR-494 and induced expression of miR-1238-3p were significantly associated with chronic brucellosis. Further research and validations are needed to evaluate the potential target genes of these miRNAs and their relation to chronicity, but through these preliminary findings we suggest that the altered expression of these four miRNAs may be novel candidate biomarkers indicating progression to chronicity.

## Figures and Tables

**Table 1:** List of significantly altered miRNA expressions in both chronic and acute brucellosis cases.

miRNA name	Chronic versus control		Acute versus control		Chronic versus acute	
	Fold regulation	*p* value^*∗*^	Fold regulation	*p* value^*∗*^	Fold regulation	*p* value^*∗*^
miR-575	9.78	0.0197	10.91	0.0267	−1.12	0.8876
miR-1914-3p	5.58	0.0557	8.27	0.0483	−1.48	0.5740
miR-339-5p	−4.11	0.1451	−4.79	0.0278	1.17	0.8520
miR-22-5p	−8.55	0.0069	−6.04	0.0258	−1.42	0.3409
miR-335-5p	−4.34	0.2607	−7.30	0.0361	1.68	0.6687

^*∗*^ *p* values were calculated using independent sample *t*-test.

**Table 2:** List of miRNAs that were differently regulated between chronic and acute brucellosis.

miRNA name	Chronic versus control		Acute versus control		Chronic versus acute	
	Fold regulation	*p* value^*∗*^	Fold regulation	*p* value^*∗*^	Fold regulation	*p* value^*∗*^
miR-125b-5p	−2.17	0.5363	−9.17	0.0115	4.22	0.2656
miR-211-3p	2.34	0.2785	5.75	0.0479	−2.46	0.0429
miR-630	3.33	0.5112	9.82	0.0450	−2.95	0.5852
miR-1290	10.89	0.1022	34.28	0.0012	−3.15	0.4310
miR-1227-5p	17.59	0.0715	78.29	0.0000	−4.45	0.2831
miR-572	9.32	0.1257	47.99	0.0002	−5.15	0.2160
miR-150-3p	2.15	0.4852	13.82	0.0042	−6.42	0.0978
miR-513b	−3.28	0.2145	2.32	0.2993	−7.61	0.0455
miR-134	4.56	0.2904	35.47	0.0016	−7.77	0.2003
miR-664b-5p	2.66	0.4824	23.36	0.0019	−8.77	0.1197
miR-125a-3p	2.48	0.4853	23.29	0.0060	−9.40	0.1125
miR-584-5p	−6.59	0.1074	2.06	0.5162	−13.57	0.0462
miR-663a	3.35	0.4647	46.77	0.0024	−13.97	0.1503
miR-1285-3p	−2.02	0.1678	7.27	0.0453	−14.70	0.0064
miR-513a-5p	−8.48	0.1474	2.54	0.3112	−21.54	0.0494

^*∗*^ *p* values were calculated using independent sample *t*-test.

**Table 3:** List of significantly altered miRNA expressions in chronic brucellosis.

miRNA name	Chronic versus control		Acute versus control		Chronic versus acute	
	Fold regulation	*p* value^*∗*^	Fold regulation	*p* value^*∗*^	Fold regulation	*p* value^*∗*^
miR-1238-3p	8.78	0.0309	−1.29	0.5934	11.35	0.0207
miR-494	−2.28	0.0516	1.08	0.7458	−2.46	0.0391
miR-6069	−3.90	0.2125	−1.19	0.6014	−12.24	0.0456
miR-139-3p	−10.49	0.0420	1.26	0.7606	−13.25	0.0283

^*∗*^ *p* values were calculated using independent sample *t*-test.

**Table 4:** List of significantly altered miRNA expressions in acute brucellosis.

miRNA name	Chronic-control		Acute-control		Chronic-acute	
	Fold regulation	*p* value^*∗*^	Fold regulation	*p* value^*∗*^	Fold regulation	*p* value^*∗*^
miR-1972	−1.23	0.8340	30.84	0.0018	−37.82	0.0040
miR-610	−1.49	0.3388	29.12	0.0048	−43.43	0.0021
miR-188-5p	1.41	0.8042	21.79	0.0165	−15.40	0.1112
miR-483-5p	1.30	0.8606	17.95	0.0464	−13.77	0.1569
miR-4793-5p	−1.13	0.9079	17.10	0.0217	−19.28	0.0322
miR-652-5p	1.18	0.8770	16.92	0.0058	−14.36	0.0466
miR-765	1.07	0.9580	14.68	0.0487	−13.73	0.1054
miR-671-5p	1.30	0.8418	13.16	0.0448	−10.16	0.1532
miR-1229-5p	1.10	0.9586	12.52	0.0333	−11.43	0.2058
miR-4257	−1.42	0.7573	11.95	0.0425	−16.94	0.0469
miR-345-3p	1.63	0.6586	11.32	0.0454	−6.93	0.1871
miR-650	−1.36	0.3388	10.61	0.0157	−14.38	0.0072
miR-622	−1.56	0.3388	10.30	0.0456	−16.10	0.0165
miR-601	−1.33	0.3388	10.00	0.0312	−13.28	0.0166
miR-1182	−1.59	0.3388	9.57	0.0490	−15.21	0.0164
miR-149-3p	1.77	0.5708	9.27	0.0353	−5.25	0.2120
miR-3135b	1.44	0.5986	9.22	0.0033	−6.40	0.0279
miR-1226-5p	−1.44	0.3388	9.06	0.0399	−13.07	0.0167
miR-659-3p	−1.43	0.3388	8.78	0.0399	−12.57	0.0168
miR-1909-5p	−1.52	0.3388	8.39	0.0476	−12.80	0.0166
miR-760	−1.41	0.3388	8.30	0.0393	−11.73	0.0167
miR-424-3p	−1.49	0.3388	7.91	0.0487	−11.79	0.0178
miR-23a-5p	−1.50	0.3388	7.79	0.0479	−11.68	0.0168
miR-665	1.46	0.6337	7.34	0.0390	−5.02	0.1529
miR-501-3p	−1.42	0.3388	6.26	0.0459	−8.88	0.0168
miR-1246	1.65	0.3648	5.65	0.0037	−3.42	0.0793
miR-574-5p	−1.05	0.9347	5.18	0.0340	−5.46	0.0520
miR-1915-3p	1.75	0.3108	4.79	0.0057	−2.74	0.1361
miR-4485	1.22	0.6117	4.32	0.0004	−3.53	0.0081
miR-638	1.65	0.4251	3.49	0.0099	−2.12	0.2898
miR-1973	−1.43	0.5252	3.41	0.0008	−4.87	0.0249
miR-4497	1.09	0.7777	3.37	0.0015	−3.09	0.0142
miR-1268a	1.46	0.4171	3.18	0.0171	−2.18	0.1773
miR-937-5p	1.27	0.4184	2.65	0.0418	−2.09	0.1325
miR-5739	−1.14	0.6948	2.32	0.0128	−2.65	0.0144
miR-4433-5p	−1.37	0.6996	−15.43	0.0213	−11.26	0.0411

^*∗*^ *p* values were calculated using independent sample *t*-test.
